# Supplementary material for: Increasing pediatric HIV testing positivity rates through focused testing in high-yield points of service in health facilities—Nigeria, 2016-2017
Source: PLoS One. 2020 Jun 19;15(6):e0234717. doi: 10.1371/journal.pone.0234717 (PMC7304582; doi:10.1371/journal.pone.0234717)
Supplement: Attachment — Submitted filename: manuscript review, pediatric HIV case finding Nigeria.docx [file pone.0234717.s002.docx]

Review Manuscript #PONE-D-19-27505

**Increasing pediatric HIV testing positivity rates through focused testing in high-yield facility-based settings—Nigeria, 2016-2017**

December 12, 2019

Dear Editor,

Thank you for the opportunity to review the article titled: ‘’ Increasing pediatric HIV testing positivity rates through focused testing in high-yield facility-based settings—Nigeria, 2016-2017’’

The authors used routinely collected data from 9 health facilities to assessed HIV seropositivity of several point of services (POS) among children 0-14 years in Nigeria. The study is relevant as it contributes information on where HIV infected children could be identified more efficiently. Overall, the problem statement is well described, the methodology sound, the results and discussion well presented. However, I believe the current version of the manuscript is not ready for publication due to the following issues:

**Title:** In the title, what is the meaning of *“high-yield facility based settings’’*? Did you mean “ high-yield point of service?’’ Somehow, the current title is not clear.

**Abstract**

1. Reading the background, the problem you intend to solve is increasing pediatric ART in Nigeria. Thus, can you consider including the contribution of each POS to HIV seropositivity. This is important to show the contribution of POPD. Actually, though POPD has a lower yield, it has a meaningful contribution in case finding.
2. In the sentence *“….group were 1-4 years =752 (41.3%); 10-14 years= 320 (17.6%);*

*5-9 years= 520 (28.5%); and <1 years=230 (12.6%)’’*, the ordering of the age group is difficult to follow. I would order the age groups from the youngest to the oldest.

3. The conclusion does not tie with the results. TB clinic has the highest yield, therefore should come first in order of priority, follow by pediatric inpatient wards and family index testing.

**Introduction**

***General comments***:

The problem statement (challenges in improving HIV case finding among children) and the efforts deploy by the Government of Nigeria (GON) and partners (notably PEPFAR) to address the issue is well described. However, there is no mentioned of the outcome implementation of the Nigeria National Acceleration Plan for Pediatric HIV treatment and Care, 2016-2018. This is the national document that introduced family index testing as a key strategy for pediatric HIV case finding in Nigeria. Thus, making reference to this Plan should further highlight the importance of the topic as well as the relevance/significance of your study. Dr. Uzoma Ene (co-author of this paper) was among the experts who elaborated this Acceleration Plan. I believe she can contribute in addressing this point.

***Specific comments:***

**Background:**

Line 4-5: I would suggest updating the figure with current UNAIDS statistics (<https://www.unaids.org/en/regionscountries/countries/nigeria>)

i) 220 000 is the upper limit of the CI. I would use but the point estimate which is 140 000 and indicate the CI= [91 000 - 220 000]
ii) 26% ART coverage is not current. Nigeria current ART coverage for children 0-14 is 35% [22 - 53].
iii) References [3] and [4] are the same (<https://www.unaids.org/en/regionscountries/countries/nigeria>). Please, delete one.

**Second to the last sentence of the 2^nd^ paragraph:**

Reference #9: please update with 2018 data: ART coverage: males= 37%; females=68% [https://aidsinfo.unaids.org].

**Last sentence of the 1^st^ paragraph:** ‘’Major reasons for the disproportionately lower coverage among children are lower HIV testing positivity rates, indicating poorly targeted testing, and lower linkage to treatment among children’’: I think lower HIV testing positivity rate is just one of the reasons for the current low pediatric ART coverage. I would revise this sentence to indicate that major reasons for the disproportionately lower coverage among children include among others lower HIV testing positivity rates.

**Methods**

Strategies implemented during PICF pilot

P.4-Please describe how the index testing modality was implemented. Is it like outpatient parents diagnosed HIV positive were invited to bring their children for testing? or were index cases recruited among parents in HIV services in hospital (e.g: ART clinic or PMTCT)? A thorough description of the implementation of the index case testing is needed to understand the processes involved and how children were recruited for testing during the intervention period.

P.5-3^rd^ sentence: ‘’Additionally, there was 100% index testing of children with HIV-positive mother or siblings’’: This sentence is confusing when reading table 2 which indicates that 85.9% of children were successful tested through family index modality. How should we understand this difference? Moreover, does it mean only children of HIV-positive mothers were tested? How about fathers who tested positive in the hospital? Were they not also ask to bring their children for testing? If no, why? What was really the entry point for family index case testing? OPD, PMTCT, ART clinic?

P.5- ‘’These PICF strategies were piloted at the TB clinics, pediatric inpatient medical wards,

adult outpatient clinic among families of identified index cases, POPDs, and the immunization clinics in the selected sites’’: where there ART and malnutrition clinics in the selected health facilities? if yes, why were they not included in the study knowing that these are high yield HIV case finding point of services?

**Results**

**P.6. Last paragraph: Age and POS were the only predictors of seropositivity assessed (Table 3). Is it possible to assess more children-level socio-demographic factors such as sex, educational level among others?**

**Table 3: I think the title should read ‘’Unadjusted and adjusted odds ratios of pediatric HIV case detection by patient age group and POS''**

**Discussion**

**2^nd^ sentence-1^st^ paragraph:** ‘’Several program strategies for improving efficiency of testing through increasing HIV positivity yield, while maintaining high numbers of HIV positive children found, and for improving efficacy of testing by assuring linkage to ART were implemented in selected health facilities providing HIV care’’.---🡪 This sentence is too long and difficult to understand. What is the message here?

**2^nd^ sentence-2^nd^ paragraph**: *‘’This may be because of a continued program emphasis on PMTCT: perhaps these youngest children were less likely to have unknown status or test positive because their mothers’ status had been confirmed during pregnancy’’---🡪* the explanation of the lowest proportion of children with unknown HIV status and tested HIV positive should be reviewed. Actually, youngest children were less likely to have unknown HIV status and test HIV positive not because ‘’ their mothers’ status had been confirmed during pregnancy’’ , but more likely because these youngest children may have been tested for HIV and those tested HIV+ received ARV prophylaxis and this through the PMTCT program. This is suggestive of the effectiveness of PMTCT program in the selected health facilities.

**4th sentence-3rdparagraph:** ‘’Even with the use of the HIV risk screening tool in the POPD, this POS gave a low yield of 1.0%’’. ---🡪 This is in my opinion a key finding of this paper considering the potential implications. Actually, the aim of the risk screening tool was to improve the yield. If this yield is still low at 1%, therefore the tool is not achieving the desired results. The reported yield in your study is even lower compared to the yield of the DHT (diagnostic HIV testing=symptoms-based testing strategy) among children reported at 4% in Cameroon ( https://journals.plos.org/plosone/article/comments?id=10.1371/journal.pone.0214251). This suggests that compared to the Bandason screening tool, better yield can even be achieved focusing on testing only HIV symptomatic outpatient children. There is urgent need to re-assess the validity of the Bandason screening tool in low HIV prevalence context such as Nigeria.

**P.8: 1^st^ sentence:** ‘’Children for HIV testing (family index testing), found that the rate of pediatric case identification increased 3.8-fold from 3.5% to 13.6% [15]’’---🡪 This statement is wrong because the reported figure correspond to the increase in HIV testing uptake and not case identification rate that was 7.4%. Please check your reference again.

**3^rd^ sentence:** ‘’Our study results were comparable with findings in these studies’’. --🡪Which of your study results are you referring to? In your study, you did not compare the HIV testing uptake (before and after the intervention) instead you reported the family index case testing HIV positivity rate of 3.5% which is far different from the 7.4% reported by Wagner et al. in Kenya. Instead, your HIV positivity rate (3.5%) corresponds exactly to the 3.5% reported in the family index case testing study in the neighbouring Cameroon by Yumo et al. (see reference#10 of your manuscript).

**2^nd^ sentence-3^rd^ paragraph**: ‘’With exception of the immunization clinic, our study found a

much higher acceptance rate among all the provider-initiated testing and counseling POS in our study sites, which may indicate that the pre-test counseling being done for families of at-risk pediatric patients at these sites is effective’’---🡪 You may support your findings with evidence from neighbouring Cameroon equally showing higher acceptance (99.7%) among parents (Yumo et al.=> see ref 10) and Ida Penda et al( .https://www.ncbi.nlm.nih.gov/pmc/articles/PMC6090739/).

**2^nd^ sentence- 4^th^ paragraph:** ‘’About 24% of children with unknown status identified through the family index POS were not tested for HIV’’----🡪This is about 2 times less than the 44% reported by Yumo et al. in Cameroon (see reference 10 of your manuscript). Can you explain this better outcome?

**4^th^ sentence-4^th^ paragraph:** ‘’Our study did not investigate the reasons for the failure of parents or guardians to return with their children’’.---🡪These reasons were investigated by Yumo et al. in a similar study in Cameroon (see reasons in ref. above).

**2^nd^ sentence-5^th^ paragraph:** ‘’Our findings that pediatric inpatient ward was an efficient POS for finding children living with HIV while maintaining a yield higher than the overall yield corroborates that guidance’’--🡪However, the contribution of inpatient (20.9%) to case finding load is similar to POPD (20.9%) and far lower to family index testing modality (55.8%). Thus, there is need for the combination of these POS, even if it means somehow losing in efficiency at the POPD. This finding simply shows the challenge in achieving optimal coverage while maintaining efficiency.

**3^rd^ sentence-5^th^ paragraph:** ‘’ We found that the family index testing modality was the most efficient POS for all sites, accounting for about 38% of total children tested but contributing more than half (55.8%) of all the positives identified’’--🡪This was the key strategy outlined in the National Acceleration Plan for Pediatric HIV Treatment and Care for Nigeria **(**Please, see Dr Uzoma Ene for reference**).** This finding indicates that if implemented at scale, this Plan could help in bridging the current gap in pediatric ART in Nigeria.

**3^rd^ sentence-5^th^ paragraph**: ‘’While we found a low yield in the POPD, because so many children seek care there in Nigeria, testing there may still represent a way to find more children living with HIV’’-🡪 This is correct, but contradicts a little bit your statement above re prioritizing inpatients testing.

**4^th^ sentence-5^th^ paragraph:** ‘’ However, in order for this to be efficient, there needs to be a program emphasis on fidelity to the HIV risk stratification tool’’-🡪 This sentence is not clear. Are you referring here to the need of using a screening tool? if yes, as indicated above, please refer to comment above re the use of the Bandason screening tool in Nigeria.

**2^nd^ sentence-6^th^ paragraph:** ‘’However, the purposive nature of site selection limits the generability of the findings from our analysis’’-🡪 Other high yield POS such ART clinics (for index case testing) and malnutrition clinics were not explored. This is another limitation of the study because information from these POS could have given more beef to the paper.

**Conclusion**

**3^rd^ sentence**: ‘’The study findings suggested that to improve facility-based HIV positivity rates

among children aged 0–14 years in Nigeria, an increased focus on HIV testing among children seeking care in pediatric inpatient wards, through family index testing, and perhaps in TB clinics is appropriate’-🡪 in this sentence, I would remove ''perhaps'', include the POPD as another priority testing point while recommending the re-assessment of the validity of the Bandason tool in low HIV prevalence context such as Nigeria. We can't afford not to test children at the OPD as it’s an important POS considering it contribution in pediatric HIV case finding load.

**Others:** The review of this paper was made unmercenary difficult because the lines are not numbered. Please, kindly numbered the lines in the revised version before re-submission.
