# Supplementary material for: Increasing pediatric HIV testing positivity rates through focused testing in high-yield points of service in health facilities—Nigeria, 2016-2017
Source: PLoS One. 2020 Jun 19;15(6):e0234717. doi: 10.1371/journal.pone.0234717 (PMC7304582; doi:10.1371/journal.pone.0234717)
Supplement: Attachment — Submitted filename: Response to reviewer 3 comments_March 17, 2020.docx [file pone.0234717.s004.docx]

Response to Reviewers’ Comments

# **Reviewer 3 Comments and Authors’ responses:**

Reviewer #3: The authors have adequately addressed my comments raised in a previous version but for reference #10 which is not well cited in line 288. Here is the correct reference: <https://doi.org/10.1371/journal.pone.0214251>. Please, note that even though this article is from the same author (Yumo et al.), it’s different from reference # 10 well cited for example in line 271. Please, amend this reference number and the references list as well.

## Authors’ response:

We have made the required changes to the reference. Please review the lines 298 and 442 to 445 of the Tracked version of the revised manuscript. The journal is now correctly cited as reference #30 in line 298 and correctly referenced in lines 442 to 445.
